# Remote Fault Diagnosis for the Powertrain System of Fuel Cell Vehicles Based on Random Forest Optimized with a Genetic Algorithm

**DOI:** 10.3390/s24041138

**Published:** 2024-02-09

**Authors:** Rui Quan, Jian Zhang, Zixiang Feng

**Affiliations:** 1 Hubei Key Laboratory for High-Efficiency Utilization of Solar Energy and Operation Control of Energy Storage System, Hubei University of Technology, Wuhan 430068, China; quan_rui@126.com (R.Q.); honey027021@163.com (Z.F.); 2 Hubei Engineering Research Center for Safety Monitoring of New Energy and Power Grid Equipment, Hubei University of Technology, Wuhan 430068, China

**Keywords:** powertrain system of fuel cell vehicle, remote monitoring, random forest, IoT platform, fault diagnosis

## Abstract

To enhance the safety and reliability of fuel cell vehicles, a remote monitoring system based on 5th generation (5G) mobile networks and controller area networks (CANs) was designed, and a random forest (RF) algorithm for the fault diagnosis for eight typical malfunctions of its powertrain system was incorporated. Firstly, the information on the powertrain system was obtained through a 5G-based monitoring terminal, and the Alibaba Cloud IoT platform was utilized for data storage and remote monitoring. Secondly, a fault diagnosis model based on the RF algorithm was constructed for fault classification; its parameters were optimized with a genetic algorithm (GA), and it was applied on the Alibaba Cloud PAI platform. Finally, the performance of the proposed RF fault diagnosis model was evaluated by comparing it with three other classification models: random search conditioning, grid search conditioning, and Bayesian optimization. Results show that the model *accuracy*, *F*1 score, and *kappa* value of the optimized RF fault classification model are higher than the other three. The model achieves an *F*1 value of 97.77% in identifying multiple typical faults of the powertrain system, as validated by vehicle malfunction data. The method demonstrates the feasibility of remote monitoring and fault diagnosis for the powertrain system of fuel cell vehicles.

## 1. Introduction

To achieve the goal of “carbon neutrality” and “carbon peak” in China, the development of green energy and electric vehicles has become a hot topic in the industry [1]. Compared to traditional oil-fueled vehicles, fuel cell vehicles are susceptible to malfunctions and safety hazards during operation due to their complex structure, poor operating conditions, strong electromagnetic interference, and uncertain external environmental factors [2,3,4,5,6]. Therefore, real-time monitoring of the vehicle’s parameters and status changes during operation, as well as timely warnings and fault diagnosis of potential faults, are essential. Existing remote monitoring technologies mainly use communication modes such as global systems for mobile communications (GSM), 3rd generation (3G), 4th generation (4G), and wireless fidelity (WiFi) to remotely transmit vehicle information data [7,8,9,10]. Although these communication modes are relatively mature and easy to implement, they cannot meet the increasing data capacity requirements of the growing network for large-scale fuel cell vehicles and have limitations in terms of real-time and reliable data transfer. In comparison, 5th generation (5G) technology provides advantages such as greater network speed, low latency, high dependability, and low power consumption, which provide technical support for intelligent networking and big data analysis of electric vehicles.

In recent years, machine learning and deep learning have made significant progress in the fault diagnosis of electric vehicles for their strong self-learning ability [11]. By learning from large amounts of data, they can automatically extract useful feature information and establish effective diagnostic models. For example, Liu et al. [12] put forward an improved machine learning-based adaptive quadratic sampling filtering FD method for multiphase drive systems. Yan et al. [13] proposed an active fault-tolerant control technique for proton exchange membrane fuel cells’ health management. Experiments showed that the method could be monitored in real-time and fault rapid diagnosis. Li et al. [14] proposed a deep learning-based diagnostic migration learning approach that uses domain adversarial training to transfer diagnostic results from suitably supervised data from several rotating machines to the target device. Wen et al. [15] proposed a two-level hierarchical diagnostic network based on a novel hierarchical convolutional neural network (HCNN), which not only models failure mode and failure severity as a hierarchy but also estimates both failure mode and failure severity. Li et al. [16] presented a system for diagnosing rolling bearing faults based on variational modal decomposition (VMD) and a modified kernel limit learning machine (KELM). The experimental findings demonstrated that the method was highly accurate. He et al. [17] proposed a brand-new hybrid deep signal processing technique for bearing defect diagnostics. The strategy created a deep learning framework with a time-synchronous resampling mechanism by combining vibration analysis techniques with deep learning. Sun et al. [18] proposed a stacked autoencoder migration learning algorithm based on class separation and domain fusion (SAE-CSDF). Zhu et al. [19] reported a new method of transfer learning (TL) based on multi-source domain adaptation. Multiple adversarial learning strategies were utilized to obtain feature representations that were invariant to multiple domain shifts while being discriminative concerning the learning target. Tian et al. [20] combined data-driven and relevant vector machine methods for the fault diagnosis of high-pressure hydrogen leakage faults in fuel cell vehicles to accurately diagnose hydrogen leakage in a short time. Gu et al. [21] presented a diagnostic method based on a long-short-term memory (LSTM) model and an embedded platform, which was proven effective in diagnosing the flooding faults of fuel cells. Yang et al. [22] proposed a current estimation method based on an artificial neural network (ANN) for single-cell short circuit faults that occurred during the charging or discharging of battery packs, and the experimental results showed that it could effectively detect the power battery faults in vehicles. Wu et al. [23] used the least squares support vector machine (LS-SVM) classifier to establish a fault diagnosis model for solid oxide fuel cells, and the findings demonstrated that the LS-SVM model could detect faults up to 97% of the time. Lim et al. [24] established an SVM model and limited data-based component-level fault diagnosis method for the thermal management system of proton exchange membrane fuel cell, and the diagnosis accuracy was 92%. Li et al. [25] provided a data-driven multi-label (ML) pattern recognition method that used feature extraction and ML-SVM classifiers to solve the diagnosis problem of simultaneous faults in solid oxide fuel cell systems. Lu et al. [26] introduced an online defect diagnostic approach for proton exchange membrane fuel cells based on rapid electrochemical impedance spectroscopy (EIS) monitoring. This technique employed a multi-fault diagnostic algorithm based on a binary tree support vector machine (DBT-SVM) classifier, and the experimental findings demonstrated that it could provide accurate and quick online fault detection of proton exchange membrane fuel cells. Lee et al. [27] used a model-based method to detect fault states with residuals greater than the threshold in the fuel cell system and then used five different classifiers (K-nearest neighbor, artificial neural network, naive Bayes classifier, and the discriminant analysis method) to classify the fault states. Test bench results demonstrated that all classifiers were able to successfully detect these faults. Zhang et al. [28] proposed a data-driven residual life prediction method that combines particle filtering, temporal attention mechanism, and bidirectional gated recurrent units. This approach integrated the strengths of data-driven and model-based methods and was validated on battery datasets. Zhang et al. [29] introduced a novel approach called the expectation maximization–unscented particle filter–Wilcoxon rank sum test (EM–UPF–W). They employed the unscented particle filter (UPF) to construct a single-cell dynamic degradation model and utilized the EM algorithm to adaptively estimate the noise variables. Additionally, the Wilcoxon rank sum test was introduced to determine the capacity regeneration point, thereby reducing prediction errors. The feasibility of this method was validated using lithium-ion battery data. Wang et al. [30] proposed a novel approach that combines a new degradation model with a particle filter to predict the health status of fuel cells. They validated the feasibility of this method using a publicly available dataset. Pan et al. [31] developed a temporal convolutional network (TCN) based on an RUL forecasting framework whose forecasting index was better than that of other models.

The fault diagnosis method based on machine learning and deep learning is an efficient, accurate, and reliable approach with advantages such as high *accuracy* and adaptivity. However, most existing studies only focus on diagnosing individual faults of fuel cell systems, and very few studies reported on the fault diagnosis of multiple faults of powertrain systems of fuel cell vehicles. Deep learning-based fault diagnosis classification methods have high computational complexity, but they are not suitable for real-time fault diagnosis environments. SVM has a dramatically increasing computational complexity with the increase in the number of features, and it requires a lot of time to learn to diagnose faults in fuel cell vehicle powertrain systems. Compared to the above methods, the random forest (RF) model is capable of handling data sets that contain redundant features and have a shorter training time. In addition, RF can quickly predict sample results, has high practicality and good real-time performance, and is very suitable for fault diagnosis and classification of complex systems [32,33]. Furthermore, RF is convenient for implementation in IoT cloud platforms. Therefore, a random forest model optimized with genetic algorithms (GA) is used for the fuel cell vehicle’s powertrain system fault diagnostics, and it is invoked on a remote monitoring and diagnostic platform developed based on the IoT platform to achieve fault prediction and diagnosis.

Traditional fuel cell systems and automotive fault diagnoses predominantly employ data-driven methods for fault classification and diagnosis, commonly known as offline diagnostics. Moreover, existing research mainly focuses on single fault diagnosis in fuel cell systems, with limited studies on multiple fault diagnosis in fuel cell automotive powertrain systems. In this study, a GA-optimized RF, combined with 5G data acquisition embedded in the Alibaba Cloud platform, was utilized for online fault diagnosis. To enable remote monitoring of fuel cell vehicles and enhance real-time, fast, and effective fault diagnosis, this research developed a remote fault diagnosis system for fuel cell automotive powertrain systems based on HUAWEI 5G communication technology and the IoT platform. Various typical faults were addressed by constructing a GA-optimized random forest fault diagnosis model on the Alibaba Cloud platform’s artificial intelligence platform. The effectiveness and practicality of this model in fault diagnosis were validated by comparing it with other algorithms. By leveraging HUAWEI 5G communication technology and the IoT platform, this remote fault diagnosis system aimed to enable efficient monitoring and timely detection of multiple faults in fuel cell automotive powertrain systems, thereby improving the real-time, fast, and effective performance of online fault diagnosis for fuel cell vehicles. The main contributions of this paper can be summarized as follows:

(1) A 5G-based cloud platform was constructed for the remote monitoring and fault diagnosis of the fuel cell vehicle’s powertrain system.
(2) A random forest model was established for the fault diagnosis of several typical faults of fuel cell vehicles.
(3) The random forest fault diagnosis model was combined with the GA algorithm and applied on an Alibaba Cloud PAI platform.
(4) The advantage of the proposed RF-GA model was validated by comparing it with the other three classical models.

## 2. Overall Architecture of Remote Fault Diagnosis System

**Figure 1:**
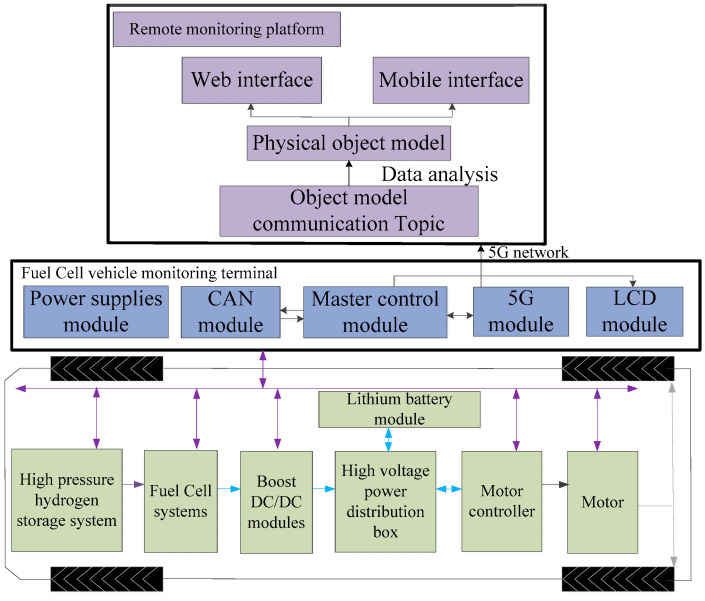
Overall architecture of remote fault diagnosis system for powertrain system of fuel cell vehicle.

The constructed remote monitoring and diagnosis system of fuel cell vehicles using 5G and IoT platforms is shown in Figure 1. It includes the powertrain system of fuel cell vehicles, 5G monitoring terminal and the remote monitoring and fault diagnosis cloud platform. The primary control module, power module, LCD display module, controller area network (CAN) communication module, and 5G module comprise the hardware of the fuel cell remote monitoring terminal. For the 5G monitoring terminal, its main control chip is an i.MX6 processor produced by NXP (Eindhoven, The Netherlands) and its power module uses the power chip of URB2405YMD-10WR3 (Mornsun, Guangzhou, China) to supply the power to the main control chip and the CAN communication module. The main body of the LCD display circuit is a 30-pin FPC base which supports the touch screen. The CAN communication module uses the ADM3053 power-isolated CAN transceiver as the communication chip to form a complete node with the integrated CAN controller in the main control chip, which is used for collecting information from the CAN bus. The 5G communication module uses the Huawei 5G industrial module of MH5000-31P (Shenzhen, China) to connect terminals to the cloud platform and upload packets, and its external interface is a Mini PCIe interface. The 5G monitoring terminal uses the CAN module to collect CAN bus messages from each control unit of the whole powertrain system, connects to the Alibaba Cloud IoT platform through 5G and message queuing telemetry transport (MQTT) protocol, and sends the messages to the IoT platform object model after processing. The remote monitoring and fault diagnosis cloud platform was developed using the Alibaba Cloud IoT platform; it includes web-side and mobile-side interfaces, which can display the real-time status information of the fuel cell vehicle. For fuel cell vehicle powertrain system malfunction data, an RF fault diagnosis model was built and deployed in the Alibaba Cloud machine learning platform PAI, which was invoked to realize real-time display and fault diagnosis.

**Figure 2:**
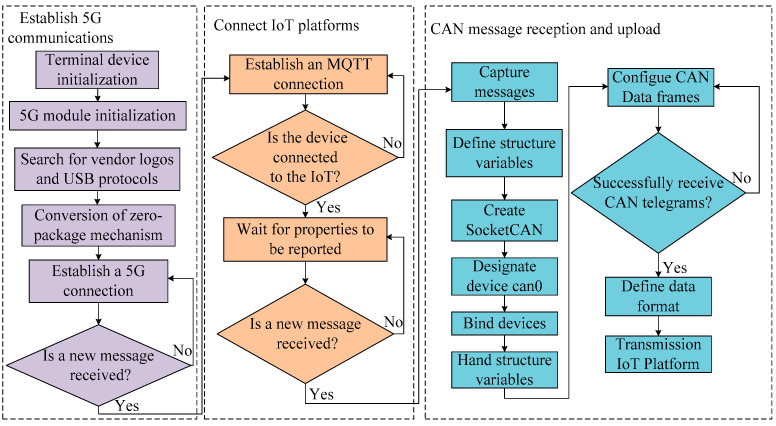
Overall flow chart of remote monitoring terminal software.

To collect and upload message information from each component of the powertrain system, a remote monitoring terminal for fuel cell vehicles was designed. A primary control module, a power supply module, an LCD module, a CAN communication module, and a 5G communication module comprise the terminal’s hardware circuit. The terminal uses the Huawei 5G industrial module MH5000-31P for cloud data uploading, and the overall flow chart of the monitoring terminal software is shown in Figure 2. Firstly, the terminal device searches for the vendor identification (Vid) and universal serial bus (USB) protocol of the module, converts the zero-packet mechanism, and starts buffering and transmitting data. After establishing 5G communication, the monitoring terminal is connected to the IoT platform based on Alibaba Cloud’s C LinkSdk and enters the waiting state to receive messages. For the monitoring terminal, it adopts SocketCAN to collect CAN messages from the powertrain system and uses relevant functions to create and bind sockets, and the message uploading to the IoT platform uses a transparent access method. As the platform will uses parsing script to parse the messages, the monitoring terminal performs binary processing and defines the structure of the received CAN messages; it encapsulates and sends the data according to the requirements of the IoT platform.

The remote monitoring and fault diagnosis cloud platform was developed based on the Alibaba Cloud IoT platform, which uses MQTT protocol to receive powertrain system messages. The platform also uses a parsing script written in JavaScript to convert the messages into Alink JSON format and store them in the object model. Additionally, the IoT Studio was utilized for the platform’s visualization development, parameter monitoring, curve and fault display of the fuel cell system, high-pressure hydrogen management unit, DC/DC, and motor and battery units in the powertrain system of the fuel cell vehicle. The interface components are linked to the object model data in the IoT platform, which enables real-time changes in various parameters, meters, signals, and curves. The optimal fault diagnosis model for the powertrain system of fuel cell vehicles based on the RF was built using custom Alink components on the Alibaba Cloud PAI platform and deployed in PAI’s model online service elastic algorithm service (EAS). The IoT platform accesses stored powertrain data and invokes the model through business logic functions to achieve a real-time diagnosis. Furthermore, the platform enables cross-platform and device interaction and enhances the accessibility and scalability of the random forest fault diagnosis model.

## 3. Remote Fault Diagnosis Model Based on RF

### 3.1. RF Algorithm

**Figure 3:**
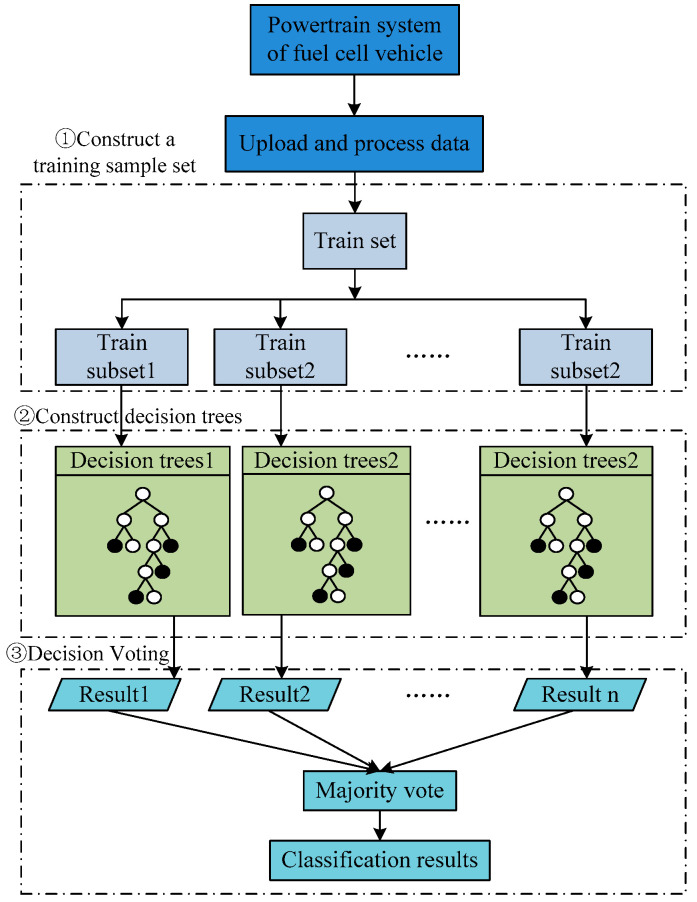
The classification process of the RF.

RF is a classification machine learning and regression approach that predicts the outcome of new data by training multiple decision trees based on a training dataset and synthesizes these decision trees [34]. For the fault diagnosis of the powertrain system of a fuel cell vehicle, random forest can be used to predict its fault type and severity. By training the model with a large amount of fault and normal data, the random forest model can identify the characteristics of various faults and predict the possible faults by combining the prediction results of multiple decision trees. The fault classification process of constructing a random forest model for the powertrain system of fuel cell vehicles is illustrated in Figure 3.

Firstly, based on the bagging idea, a portion of fault data is randomly selected from the fault dataset of the powertrain system as a sub-training set *D_i_* (*i* = 1, 2, 3, …, n) for each tree. These data are selected through random sampling with replacement to avoid overfitting and improve the model’s generalization ability. To construct each tree, fault features of the powertrain system need to be randomly extracted to obtain a subset of features for the current tree.

Then, for each partitioned training set, the classification and regression tree (CART) technique is used to create a decision tree and determine the appropriate splitting points of the tree nodes utilizing information gain, which is a metric used to measure the quality of the split and represent the difference between the uncertainty before and after the split [35]. The larger the information gain, the smaller the uncertainty after the split, which indicates better split quality. The information entropy of the sub-training set corresponding to the current node is calculated as follows:

(1) $$H\left(D_{i}\right)=-\sum P(C_{j})\times \log_{2}(P(C_{j})),$$

where *H*(*D_i_*) represents the information entropy of the sub-training set corresponding to the current tree, and *P*(*C_j_*) is the probability of occurrence of fault type *C_j_* in *D_i_*, which can be written as follows:

(2) $$P(C_{j})=\frac{count(C_{j})}{\left|D_{i}\right|},$$

where *nt* (*C_j_*) stands for the number of occurrences of fault type *C_j_* in *D_i_*, and |*D_i_*| denotes the total number of samples in *D_i_*.

For each fault feature *A*, the information gain obtained after splitting based on it (denoted Gain (*D_i_*, *A*)) [36] is calculated as follows:

(3) $$Gain(D_{i},A)=H(D_{i})-H(D_{i}\;|\;A),$$

where *H* (*D_i_* | *A*) means the information entropy after division according to the fault feature *A*, which indicates the uncertainty of different fault categories in each subset after division.

The specific calculation process is as follows:

(4) $$H(D_{i}\;|\;A)=-\sum \left(P(X)\times \sum P(C_{j}\;|\;X)\times \log_{2}(P(C_{j}\;|\;X))\right),$$

where *P*(*X*) denotes the probability of the subset of fault features *A* taking value *X* in the training set, and *P* (*C_j_* | *X*) indicates the probability of occurrence of fault type *C_j_* in the subset.

The CART tree selects the fault feature with the greatest information gain as the splitting feature of the current node, divides the data set into several subsets according to the value of the dividing feature, and recursively performs the above steps for each subset until the stopping condition is satisfied. On this occasion, the final decision tree construction is completed.

Finally, the random forest uses majority voting to determine the final fault diagnosis results, and the particular voting procedure is as follows:

(1) For each decision tree, the fault diagnosis result of each tree is obtained based on the input fault data.
(2) Count the occurrence number of each fault type in all diagnostic results.
(3) Select the fault type with the highest occurrence number as the final fault diagnosis result.

For the fault type *C_j_* and the trained n decision trees [37], the prediction result of the random forest can be written as follows:

(5) $$C_{j}=\arg\max(\sum T(i)=C_{j})(i=1,2,3\ldots n),$$

where *T*(*i*) denotes the diagnostic result of the ith tree, ∑*T*(*i*) = *C_j_* indicates the number of diagnostic faults of type *C_j_* in all decision trees, and arg max is the subscript corresponding to the maximum value in a set of values.

### 3.2. GA Algorithm

**Figure 4:**
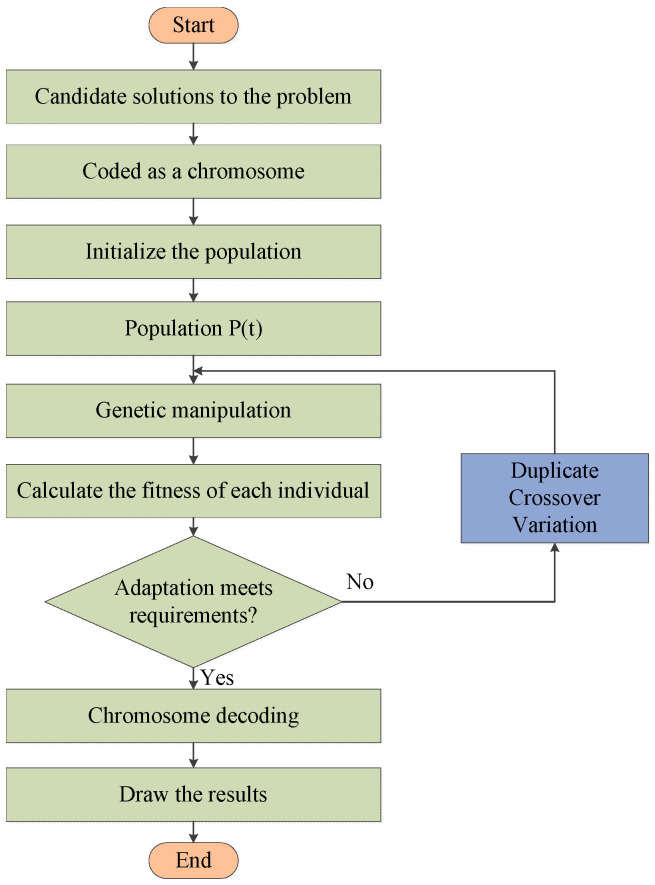
The flowchart of GA.

The GA possesses inherent, implicit parallelism and superior global optimization capabilities. It can handle multiple individuals within a population, making them less prone to becoming trapped in local optima. Moreover, GA exhibits self-organization, self-adaptation, and self-learning abilities. The genetic algorithm process includes initializing the population, evaluating the fitness of individuals, selection operation, crossover operation, mutation operation, etc. In genetic algorithms, individuals in the population are treated as solutions to the problem. The algorithm starts by randomly generating initial individuals in the population. Then, based on the defined fitness criteria, it selects individuals with higher fitness, which is more in line with the optimal solution to the problem. These selected individuals undergo crossover, mutation, and other operations to generate the individuals of the next generation. The process of evaluating fitness, selection, and other operations is repeated iteratively, updating the population until the optimal solution is found. The flowchart of the GA is shown in Figure 4. The GA first encodes the parameters to be solved, forming a population similar to chromosomes, which can undergo genetic operations. It then simulates the processes of selection, crossover, and mutation in biological genetics while preserving individuals with the best fitness values. After multiple iterations of genetic operations, the final optimal solution is obtained [38]. The GA solves problems through random searching of the population, allowing optimization of the entire population and avoiding becoming stuck in local optima. It performs well in highly nonlinear systems and has better adaptability, convergence speed, and effectiveness. The crossover probability is 0.7, and the population size is 50.

### 3.3. Optimization of RF Model

**Figure 5:**
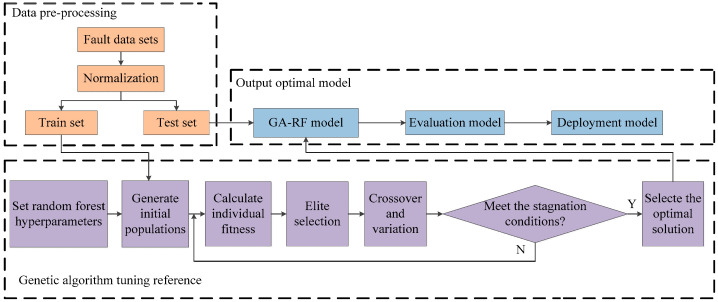
Fault diagnosis flowchart of GA–RF.

To construct an RF model, several hyperparameters need to be determined, and these hyperparameters have an important impact on the *accuracy* and efficiency of the model. Therefore, appropriate hyperparameters are the key to improving the fault diagnosis effect of RF models. In this study, different RF models are generated, GA is combined to optimize their hyperparameters, and then the *accuracy* and efficiency of the models are improved. The flow of the RF fault diagnosis model optimized by GA (denoted as GA–RF) for the powertrain system of the fuel cell vehicle is shown in Figure 5.

**Figure 6:**
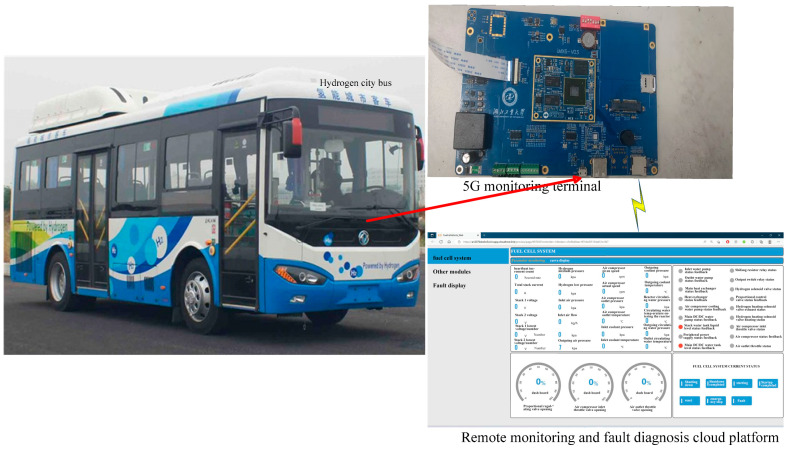
EQ6850CACFCEV fuel cell bus.

Firstly, a fault dataset is obtained for data preprocessing. Typical faults that can affect the powertrain system performance of fuel cell vehicles and make it impossible for them to drive normally include DC/DC working abnormalities, motor failures, and stack film drying and flooding. Lithium battery insulation failures and internal short circuits can lead to battery safety hazards and even combustion in serious cases. Faults such as hydrogen gas pipe leakage and cooling system failure can affect vehicle safety and cause serious accidents. Therefore, considering the impacts of various faults on the powertrain system of fuel cell vehicles, eight typical faults shown in Table 1 are selected, and the corresponding fault characteristics shown in Table 2 reflect the changes of faults for model training and evaluation. Furthermore, the data used to establish the model are obtained from the actual measurement data of an 8.5 m fuel cell bus (type EQ6850CACFCEV) developed by a domestic company, which is shown in Figure 6; Table 3 shows the major parameters of this fuel cell bus. The fuel cell bus has run over 8000 km on the road between Laohekou City and Wuhan City in China, and it includes a total of 2500 sets of the mentioned fault types during three years of demonstration operation.

Due to the potential impact of features with larger ranges of failure values on the splitting process of decision trees, this paper proposes feature normalization to ensure a relatively balanced contribution from each feature to the model. This approach aims to prevent certain features from dominating the training process and to ensure that all failure features have similar importance and scale. Considering that the data have different scales and ranges, a common min–max normalization is used to map the value range of the original fault data to between 0 and 1, and the specific formula is as follows:

(6) $$x_{n}=\frac{\left(x-x_{\min}\right)}{\left(x_{\max}-x_{\min}\right)},$$

where *x_n_* represents the normalized value of the fault feature, *x* represents the original value of the fault feature, and xmin and xmax mean the minimum and maximum values of the fault feature in the dataset, respectively. Then, the normalized dataset is split into a 7:3 ratio for training and evaluation testing of the RF model, respectively.

To optimize the random forest model using the GA, the important hyperparameters that need to be tuned in the RF model are first determined, such as the number of trees in the RF and the depth of a single tree; these parameters are defined as the parameter space. Then, some parameter combinations are randomly generated in the parameter space as the initial population, and a given test set of failure data is used to construct the random forest model corresponding to each population. Subsequently, each individual is evaluated to obtain the model performance of each parameter combination. Finally, models with higher fitness are selected for crossover and mutation of the population, and the newly generated individuals are added to the population and used to replace the individuals with lower fitness. The above steps are repeated until the maximum number of iterations is obtained or the optimal solution is found, and the optimal RF model is then selected from the final population.

**Figure 7:**
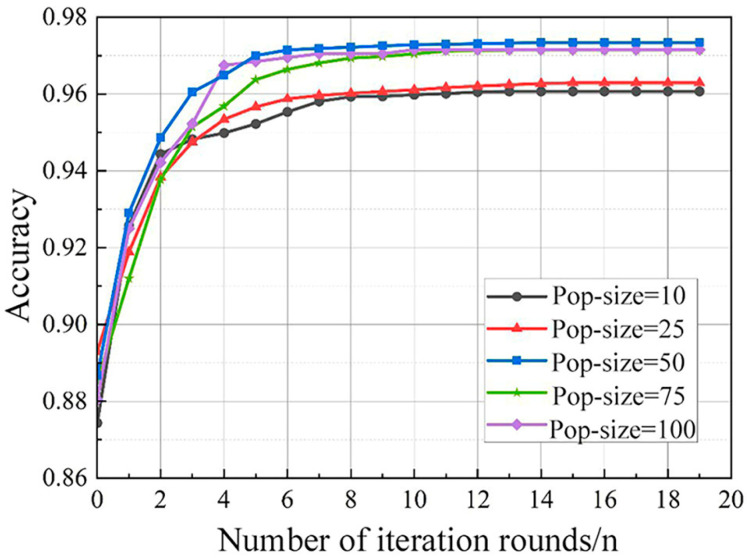
Effect of population size on model accuracy.

**Figure 8:**
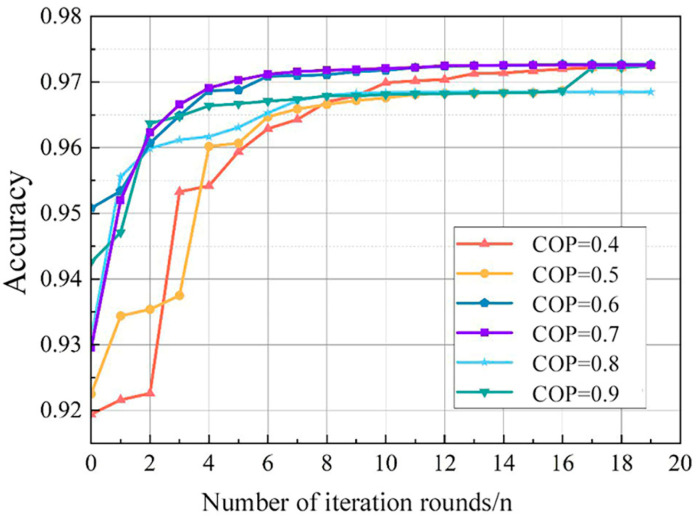
Effect of crossover probabilities on model *accuracy*.

Considering the population size (pop size) directly affects the algorithm performance and search effectiveness, and the crossover probability (COP) determines the probability of recombination among individuals. Pop size and COP are adopted to adjust the parameters of the RF model by comparing the *accuracy* of the random forest model with different crossover probabilities. Figure 7 and Figure 8 indicate the influence of these variables on model *accuracy*. It can be seen that a pop size of 50 can achieve a better tuning effect, and the model *accuracy* converges smoothly with a COP of 0.7, which is better than other cop settings.

**Figure 9:**
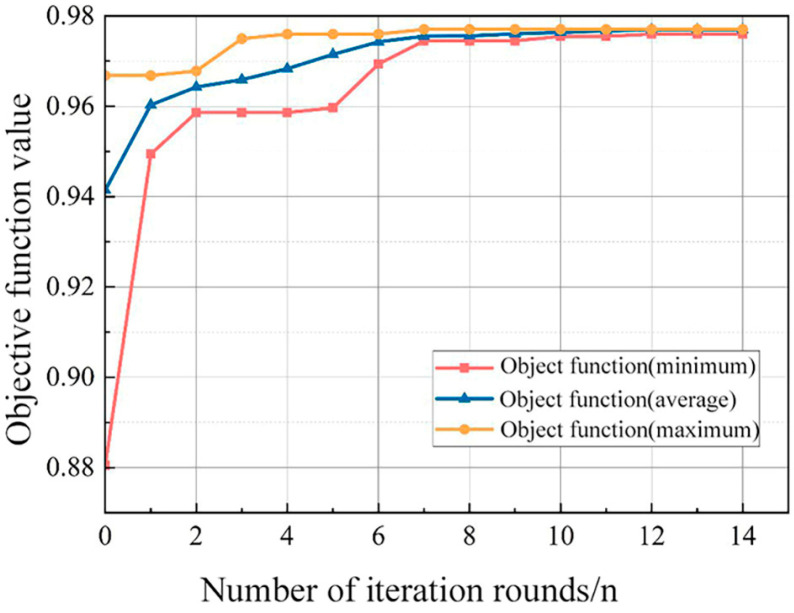
Effect of the iteration number on the objective function values.

The final selected parameters for the GA are as follows: population size is 50, crossover probability is set to be 0.7, the maximum number of iterations is 100, stagnation judgment threshold is 10^−6^, and the objective function is *F*1-macro. The GA-based tuning iteration is shown in Figure 9. It can be observed that the minimum, average, and maximum objective function values increase with the increasing iteration number, reach a stable value at the 14th generation, and then the iteration stops. In this case, the optimal parameters of the RF model are shown in Table 4.

### 3.4. Model Evaluation

To evaluate the proposed RF model, three evaluation metrics such as model accuracy (*Accuracy*), *F1* value, and *kappa* value [39] are calculated. Among them, model *accuracy* refers to the ratio of the number of samples correctly predicted by the model to the total number of samples, and it can be expressed as follows:

(7) $$Accuracy=\frac{TP+TN}{TP+TN+FP+FN},$$

where *TP* denotes the number of positive samples predicted correctly, *TN* denotes the number of negative samples predicted correctly, *FP* stands for the number of samples predicted to be positive that are actually negative, and *FN* is the number of samples predicted to be negative that are actually positive.

The *F*1 value represents the harmonic mean of precision and recall, and a higher *F*1 value indicates that the model performs well in both precision and recall.

(8) $$F1=\frac{2\times (Pre\times Rec)}{Pre+Rec},$$

where *Pre* indicates the proportion of samples predicted to be positive that are actually positive, and *Rec* means the proportion of samples predicted to be positive that are actually positive.

The *kappa* value indicates the correlation between the predicted and true results of the model. It can be expressed as follows:

(9) $$kappa=\frac{po-pe}{1-pe}$$

where *po* is the ratio of the number of correctly predicted samples to the total number of samples in the true results, and *pe* denotes the coincidence of the predicted and true results in each category.

Table 5 lists the evaluation values of the three metrics of the optimal RF fault diagnosis model. It can be seen that the values of all three metrics are above 0.97, which indicates that the fault diagnostic model has a low misclassification rate, a good balance between precision and recall, and a high consistency between predicted and true categories. In all, the model exhibits a high ability to correctly predict sample categories.

### 3.5. Deployment and Invocation of RF Fault Diagnosis Model

To construct the optimal RF fault diagnosis model, it is deployed through the Alibaba Cloud PAI platform. Multiple steps of the machine learning task are combined into a machine learning pipeline by customizing the Pipeline class in the Alink component, and each operation within the pipeline processes data and trains the model in sequence. Finally, the trained model is deployed as an online service using the EAS function to implement a real-time intelligent fault diagnosis function.

**Figure 10:**
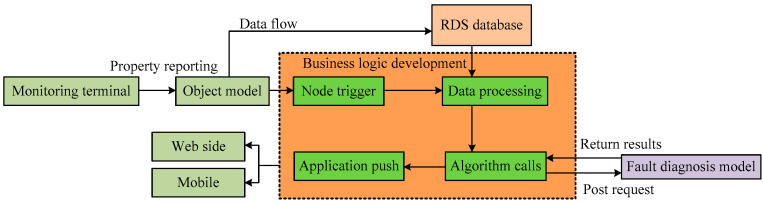
Business logic node flow.

Moreover, a remote fault diagnosis scheme for the powertrain system of fuel cell vehicles is proposed using the business logic development tool of IoT Studio. The business logic node flow is depicted in Figure 10, which includes device triggering, data processing, algorithm invocation, and application push. In the data processing stage, a JavaScript script node is used to process the data and ensure it meets the input format requirements of the model. In the algorithm invocation stage, a POST request is sent to invoke the deployed random forest fault diagnosis model in the cloud and obtain the diagnostic results. In the application push stage, the diagnostic results are pushed to a visualization application to display the fault outcomes. In the fault diagnosis interfaces on both the web and mobile platforms, within the text component and signal light component configuration panels, selecting application push as the data source allows access to the pushed information. Subsequently, the data filtering script utilizes the filter () function to filter out fault results that do not comply with the component properties, enabling the display of corresponding component fault results.

## 4. Results and Discussion

### 4.1. Model Evaluation Metrics with Different Optimization Algorithms

**Figure 11:**
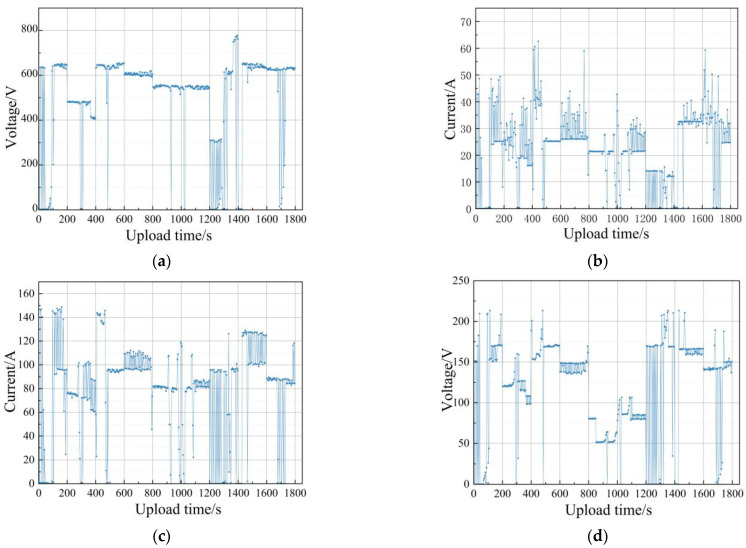

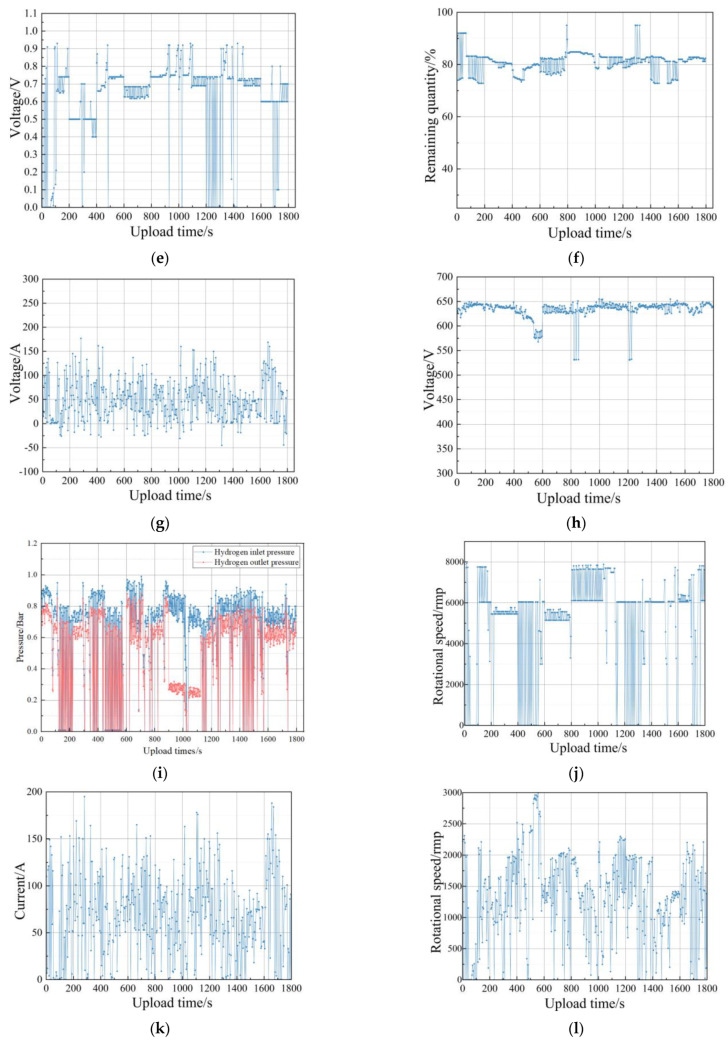

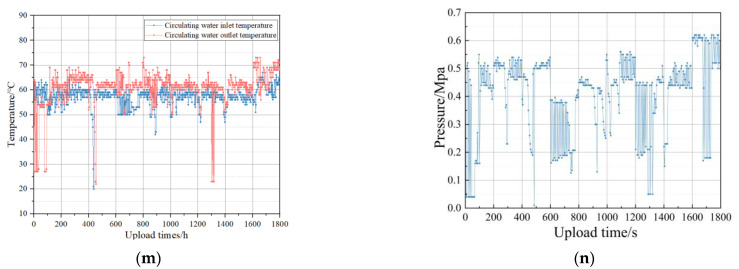
Measured fault feature curves. (**a**) DC/DC output voltage. (**b**) DC/DC output current. (**c**) Total stack current. (**d**) Total stack voltage. (**e**) Minimum monolithic voltage of the stack. (**f**) Lithium battery SOC. (**g**) Lithium battery current. (**h**) Lithium battery voltage. (**i**) Hydrogen inlet and outlet pressure. (**j**) Air compressor speed. (**k**) Motor current. (**l**) Motor speed. (**m**) Circulating water inlet and outlet temperature. (**n**) Circulating water pressure.

A total of 450 sets of fault data recorded from the actual operation of the aforementioned fuel cell bus are selected for comparative analysis of the model, which includes a normal type and eight fault types, and 50 sets of data are selected for each type. Some typical fault feature curves are shown in Figure 11, and the constructed model is compared with other RF models optimized using several different algorithms and other decision tree classification models to verify its superiority.

Based on the above methods, RF models are constructed using three different optimization algorithms: random search conditioning (denoted as RS–RF), grid search conditioning (denoted as GS–RF), and Bayesian optimization (denoted BO–RF). For a random search, the number of search iterations (n_iter) is set to 500, which means the best model is selected after 500 searches. For a grid search, 10 cross-validation folds (CV) are applied. For the Bayesian optimization algorithm, the number of search iterations (n_iter) is set as 20, the number of initialization points (n_initial_points) is 10, and the expected improvement (acq_func = ‘EI’) is used as the search strategy.

The comparison results of the RF model optimized with random search, grid search, Bayesian optimization, and the GA are shown in Table 6. As shown in Table 6, the results of the four algorithms are very similar when adjusting the bare minimum of data necessary for splitting and leaf nodes, and their differences mainly lie in the number of decision trees and the maximum number of leaf nodes.

**Figure 12:**
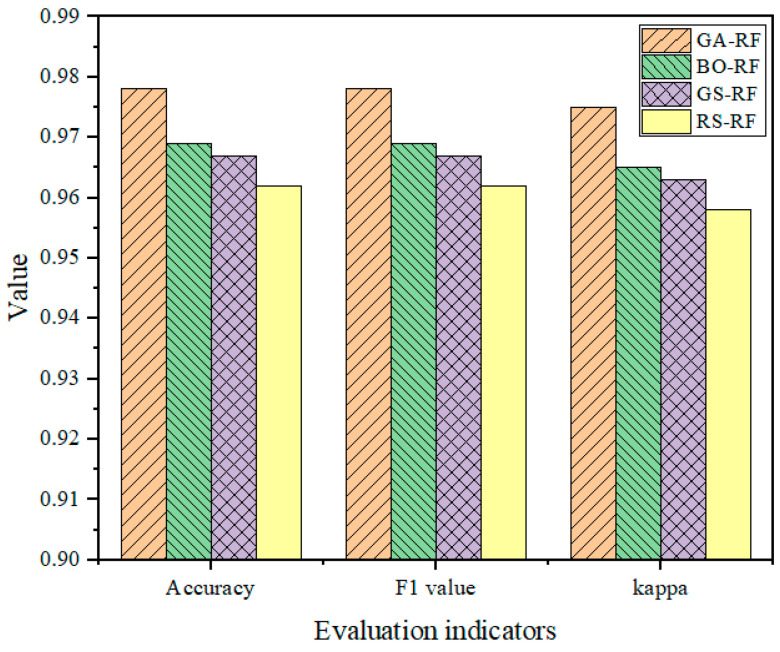
Comparison of evaluation metrics for RF models optimized with different optimization algorithms.

The comparison results of the four RF models optimized with the above four different optimization algorithms are shown in Figure 12. Obviously, RS–RF has the worst performance and ranks last in each index. The reason is that the search process of the random search algorithm is relatively arbitrary compared to other tuning methods, and it does not consider the correlation among parameters, which results in a suboptimal combination of hyperparameters that affects the model’s performance. In contrast, the performance of the BO–RF and GS–RF models is similar but still inferior to that of the GA–RF model. The GA–RF model has the best performance in all indexes, which indicates that GA has the advantages of global search capability, adaptive search strategy, and parallelization, and it can determine the optimal combination of model parameters and improve the fault diagnosis RF model’s performance.

### 4.2. Fault Diagnosis Performance of Different RF Models

To further validate the fault diagnosis performance of the proposed GA–RF model, it is compared with several other typical machine learning classification models, such as extreme gradient boosting (XGBoost), gradient boosting decision tree (GBDT), and adaptive boosting (Adaboost). The optimal hyperparameters for the XGBoost, GBDT, and Adaboost models are shown in Table 7. By utilizing a genetic algorithm to optimize these hyperparameters, the aim was to minimize errors and improve overall performance. This indicates that the selected hyperparameters have the potential to yield better model performance compared to default or randomly chosen hyperparameters.

**Figure 13:**
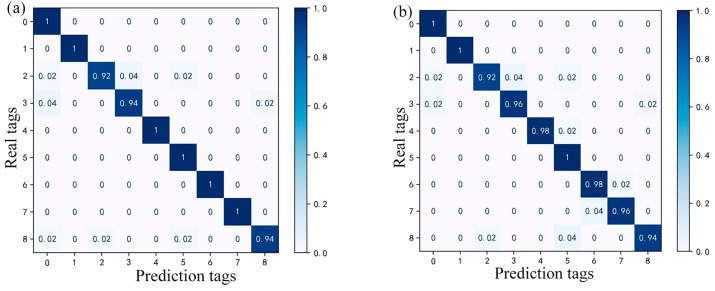

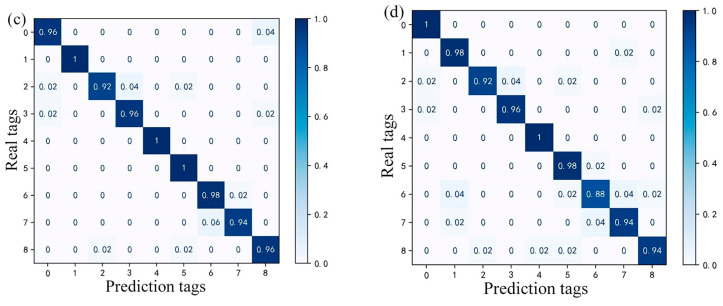
Confusion matrix of different RF fault diagnosis models. (**a**) GA–RF, (**b**) GA–XGBoost, (**c**) GA–GBDT, and (**d**) GA–AdaBoost.

To visually verify the fault diagnostic performance of each model, the above-mentioned 450 groups of fault data are tested, and Figure 13 shows a visualization of the confusion matrix.

It can be observed that the constructed RF fault diagnostic model in this study is better compared to the other three, and only three fault types have a small number of misclassifications, which may be caused by the imbalance of the training data. Specifically, for faults labeled 6 and 7 (motor fault and DC/DC operation abnormality), the other three RF models produced a certain degree of misclassification due to the similarity of some fault features, but the proposed GA–RF model predicted them correctly. Therefore, when similar features exist, the traditional decision tree algorithm may suffer from overfitting, whereas the RF algorithm can reduce the influence of similar features when setting up the decision tree by randomly selecting features; thus, its generalization ability is improved.

Furthermore, the comparative results of each model regarding the above three evaluation metrics are shown in Table 8. The results indicate that the overall fault classification performance of the GA–RF model is superior to the other three models. The *accuracy* of XGBoost, GBDT, and Adaboost is 0.9733, 0.9689, and 0.9552, respectively, while the *accuracy* of GA–RF is 0.9767, which is higher by 0.0034, 0.0078, and 0.0215 compared to the other three models, respectively. The *F*1 scores of XGBoost, GBDT, and Adaboost are 0.9733, 0.9689, and 0.9556, respectively, while the *F*1 score of GA–RF is 0.9761, which is higher by 0.0028, 0.0072, and 0.0205 compared to the others, respectively. The *kappa* values of XGBoost, GBDT, and Adaboost are 0.9729, 0.9679, and 0.9535, respectively, while the *kappa* value of GA–RF is 0.9733, which is higher by 0.0004, 0.0054, and 0.0198 compared to the other three models, respectively. The advantage in *accuracy* demonstrates that the proposed GA–RF model can diagnose faults in fuel cell vehicle powertrain systems more accurately. The comparison of the *F*1 score indicates that the proposed GA–RF model is better at identifying positive samples and can balance *accuracy* and recall. The comparison of the *kappa* value validates that the proposed GA–RF model has better resistance to random errors.

## 5. Conclusions

This study developed a remote monitoring and fault diagnostic system based on 5G technology and an IoT cloud platform to accomplish remote monitoring and problem detection of the powertrain system of a fuel cell car. The system achieved the collection, uploading, and monitoring of message data of the powertrain system and deployed an RF-based fault diagnosis model using the PAI platform. This breakthrough overcame the remote monitoring and fault diagnosis limitations of traditional vehicles.

The important hyperparameters of the RF fault diagnosis model were optimized using GA, which effectively improved the model’s comprehensive capability. The comparative findings revealed that the developed GA–RF diagnosis model efficiently identified eight common problem types in a fuel cell vehicle’s powertrain system, and its fault diagnosis performance was better than that of the XG Boost and GBDT models with higher *accuracy*, *F*1 values, and *kappa* values.

The GA–RF model deployed in the cloud platform can not only process high-dimensional and sparse fault data but it also effectively handles noise and high-dimensional fault data, which is very advantageous in the fault diagnosis of fuel cell vehicles with a large amount of data and is practical to the application.

## Figures and Tables

**Table 1:** Fault type and label.

Fault Type	Failure Tags
Normal state	Normal
DC/DC working abnormality	Fault1
Lithium battery insulation failure	Fault2
Air compressor failure	Fault3
Hydrogen leak	Fault4
Cooling system failure	Fault5
Electric stack film dry	Fault6
Flooding of power pile	Fault7
Motor failure	Fault8

**Table 2:** Fault feature.

Feature Name	Feature Tags	Feature Unit
DC/DC output terminal current	DCHC	A
DC/DC output terminal voltage	DCHV	V
Total stack current	FcCure	A
Total stack voltage	FcVolt	V
Minimum single voltage of the fuel cell stack	FcVoltPer	V
Hydrogen inlet pressure	H_2_In	Bar
Hydrogen outlet pressure	H_2_Out	Bar
Lithium battery temperature	LiT	°C
Lithium battery current	LiCure	A
Lithium battery SOC	LiSoc	-
Lithium battery voltage	LiVolt	V
Motor current	MotorC	A
Motor speed	MotorSpeed	Rmp
Air compressor speed	ACSpeed	Rmp
Inlet stack air pressure	ACIn	Bar
Circulating water pressure	WaterPIn	MPa
Temperature of reactor circulating water inlet Intlet coolant temperature	WaterTIn	°C
Outlet coolant temperature	WaterTOut	°C

**Table 3:** The main parameters of the EQ6850CACFCEV.

Parameters	Failure Tags
Wheelbase	4200 (mm)
Axial load	4500/4800 (mm)
Front suspension/rear suspension	1920/2350 (mm)
Maximum speed	69 (km/h)
Power	127 (kw)
Horse power	172 (ps)

**Table 4:** Parameters of optimal RF model.

Parameter Name	Parameter Value
Number of decision trees	330
Minimum number of split samples	5
Minimum sample size of leaf nodes	2
Maximum number of leaf nodes	173

**Table 5:** GA–RF model evaluation metrics.

Indicators	Values
*Accuracy*	0.9767
*F*1	0.9733
*kappa*	0.9761

**Table 6:** Optimization results for different algorithm parameters.

	GA–RF	RS–RF	GS–RF	BO–RF
Number of decision trees	330	390	280	240
Minimum number of samples required for splitting	5	4	2	5
Minimum number of samples required for leaf nodes	2	1	1	2
Maximum number of leaf nodes	173	148	147	120

**Table 7:** Hyperparameter setting for each model.

Models	Hyperparameter Setting
XGBoost	Number of decision trees: 200; Learning Rate: 0.3; Minimum leaf weights: 1; Maximum depth: 9
GBDT	Number of decision trees: 160; Learning Rate: 0.2; Maximum depth: 4; Maximum number of features: 5; Minimum number of samples required for a leaf node: 5
Adaboost	Weak Learners: CART Tree; Number of weak learners: 200; Learning Rate: 0.2

**Table 8:** Comparison of diagnostic performance of each model.

Evaluation Indicators	GA–RF	XGBoost	GBDT	Adaboost
*Accuracy*	0.9767	0.9733	0.9689	0.9552
*F*1	0.9761	0.9733	0.9689	0.9556
*kappa*	0.9733	0.9729	0.9679	0.9535

## Data Availability

The data presented in this study are available on request from the corresponding author.
